# Application of Omics Analyses in Pediatric B-Cell Acute Lymphoblastic Leukemia

**DOI:** 10.3390/biomedicines13020424

**Published:** 2025-02-10

**Authors:** Megi Vllahu, Maria Savarese, Immacolata Cantiello, Carmen Munno, Rosalba Sarcina, Pio Stellato, Ornella Leone, Mariaevelina Alfieri

**Affiliations:** 1 Department of Precision Medicine, Università of Campania “Luigi Vanvitelli”, 80138 Naples, Italy; megivllahu90@gmail.com; 2 Clinical Pathology, Santobono-Pausilipon Children Hospital, 80129 Naples, Italy; m.savarese@santobonopausilipon.it (M.S.); i.cantiello@santobonopausilipon.it (I.C.); c.munno@santobonopausilipon.it (C.M.); r.sarcina@santobonopausilipon.it (R.S.); o.leone@santobonopausilipon.it (O.L.); 3 Oncohematology Unit, Department of Oncology, Hematology and Cellular Therapies, Santobono-Pausilipon Children Hospital, 80129 Naples, Italy

**Keywords:** cancer, B-ALL, pediatric leukemia, lymphoblastic leukemia, genomic analysis, omics, NGS, RNA-seq, WGS

## Abstract

Acute lymphoblastic leukemia (ALL) is the most common pediatric cancer, comprising almost 25% of all malignancies diagnosed in children younger than 20 years, and its incidence is still increasing. ALL is a blood cancer arising from the unregulated proliferation of clonal lymphoid progenitor cells. To make a diagnosis of B-cell ALL, bone marrow morphology and immunophenotyping are needed; cerebrospinal fluid examination, and chromosomal analysis are currently used as stratification exams. Currently, almost 70% of children affected by B-cell ALL are characterized by well-known cytogenetic abnormalities. However, the integration of results with “omic” techniques (genomics, transcriptomics, proteomics, and metabolomics, both individually and integrated) able to analyze simultaneously thousands of molecules, has enabled a deeper definition of the molecular scenario of B-cell ALL and the identification of new genetic alterations. Studies based on omics have greatly deepened our knowledge of ALL, expanding the horizon from the traditional morphologic and cytogenetic point of view. In this review, we focus our attention on the “omic” approaches mainly used to improve the understanding and management of B-cell ALL, crucial for the diagnosis, prognosis, and treatment of the disease, offering a pathway toward more precise and personalized therapeutic interventions.

## 1. Introduction

Acute lymphoblastic leukemia (ALL) is the most common cancer of childhood, accounting for 21% of diagnoses among children from birth to 14 years of age and 7% of diagnosed cases among adolescents (aged 15–19 years) [1]. The rate of new cases was 4.9 per 100,000 children per year based on 2016–2020 cases. Approximately 53.5% of ALL cases occur in children and adolescents younger than 20 years, and it is more frequent in boys than in girls, with a ratio of 1.25:1. ALL arises from the malignant transformation of lymphoid progenitor cells in the bone marrow. This process is driven by the accumulation of genetic mutations and chromosomal abnormalities in multiple clonal populations of blast precursors [2]. The clonal heterogeneity observed in ALL suggests that multiple competing clones can evolve simultaneously, with selective pressures during disease progression and treatment shaping the dominant cancer phenotype. This dynamic clonal architecture contributes to both initial disease development and relapse, highlighting the complexity of ALL [3]. Based on immunophenotyping, ALL is classified as B-ALL or T-ALL. About 85% of pediatric cases are represented by B-ALL [4].

**Figure 1:**
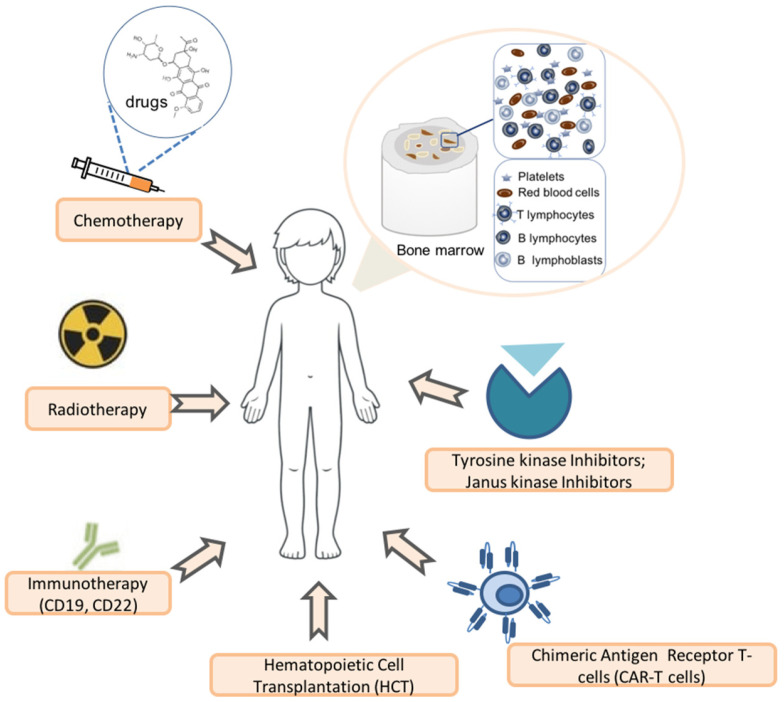
Current therapeutic options for acute lymphoblastic leukemia in children.

B-ALL originates from immature B-cell precursors and is characterized by the expression of markers such as CD19, CD10 (common acute lymphoblastic leukemia antigen), and terminal deoxynucleotidyl transferase (TdT), reflecting the immaturity of the precursor cells. ALL stands as a model for successful childhood cancer treatment. Over the past sixty years, the survival rate has increased from approximately 10% in 1960 to over 85–90% today, thanks to the advancements in risk stratification and tailored therapy approaches. The overall complete remission rate in children exceeds 95%. Nevertheless, approximately 20% of children with B-ALL still experiences relapse, which is associated with a less favorable prognosis [5]. Traditionally, genetic factors identified through conventional karyotyping have served to diagnose ALL and stratify the risk faced by children affected by the disease [6]. The integration of minimal residual disease monitoring further refines risk assessment and treatment adjustment. However, the conventional chemotherapy approach has been raised to the limit, and further improvements in outcomes will require novel biologically targeted therapeutic strategies. Currently, therapeutic protocols for pediatric ALL combine conventional approaches, such as chemotherapy and radiotherapy, with novel immunotherapeutic agents (e.g., blinatumomab and inotuzumab) targeting surface antigens, alongside targeted treatments using kinase inhibitors for specific ALL subtypes. In recent years, a novel immunological approach based on chimeric antigen receptor T-cells (CAR-T cells) has been developed and introduced for the therapy of relapsed patients, showing great promise for this complex subset of patients [7,8,9,10] (Figure 1).

With advancements in molecular diagnostics, risk stratification became a cornerstone of tailoring therapy for the identification of patients who require intensive treatment versus those who may benefit from less aggressive approaches to minimize long-term side effects.

A good or poor prognosis can be predicted on the basis of different factors reported in Table 1 [5,11].

This review highlights the transformative potential of “omic” techniques, which analyze thousands of biomolecules simultaneously, for uncovering deeper molecular insights into B-ALL. By focusing on the role of “omic” technologies, this review underscores their importance in advancing the understanding and management of B-ALL, offering a pathway toward more precise and personalized therapeutic interventions.

## 2. The Advent of Omics Analyses in Acute B-Cell Lymphoblastic Leukemia

Risk-adapted therapies may strongly contribute to the improvement of survival rates in pediatric acute lymphoblastic leukemia (ALL); therefore, the detection of chromosomal aberrations is mandatory for risk stratification.

In recent years, genomic analyses and especially transcriptome sequencing, have made it possible to identify multiple new subtypes not evident on cytogenetic analysis due to cryptic rearrangements or sequence mutations. Of course, these recent advances in molecular diagnostic technologies have improved the accuracy of risk stratification, and pave the way to achieving personalized treatments and better clinical outcomes. We have summarized the main aspects of these new molecular approaches used in the diagnosis of B-ALL.

### 2.1. Next-Generation Sequencing

Standard of care (SoC) techniques for clinical identification of B-ALL include cytogenetic analysis, FISH, RT-PCR, MLPA, and SNP-arrays. When standard diagnostic screening fails to detect disease-defining or risk-stratifying lesions, patients are classified as B-other ALL, emphasizing the importance of genomic markers in precise diagnosis and personalized strategies. Using current techniques, the accurate detection of genetic abnormalities is challenging, due to the rapidly expanding aberration list. Integrating next-generation sequencing (NGS) into the clinical management of ALL patients reveals several novel molecular entities, enhancing understanding of existing ones and identifying prognostically significant subtypes, crucial for adults lacking childhood ALL markers.

Upon developing a customized NGS panel, Montaño et al. demonstrated its efficiency, accuracy, and ability to detect key B-ALL genetic alterations in a single step within 3 h per sample, exhibiting concordance with standard techniques for over 90% of alterations as point mutations, structural rearrangements, and copy number variations [12].

Recent studies have explored the application of more extensive NGS platforms (whole exome, whole genome, and trascriptome) to investigate clinical cancer profiling.

Combined genomic and transcriptomic features reveal up to 23 subtypes of B-ALL, some of which are more common or rare depending on the age of the patients [13]. The revised taxonomy of B-ALL subtypes improved the diagnosis, prognosis, and treatment of the disease. The genomic classification enhances risk assignment in adult B-ALL patients, compared to the conventional risk factors, stratifying patients into standard, intermediate, and high-risk groups based on their subtypes [14].

Third-generation sequencing revolutionizes DNA and RNA analysis with long reads up to 1 Mb, ideal for studying large structural variations in cancer. Among these, nanopore sequencing with its rapid and targeted identification of fusion oncogenes offers real-time analysis within just 5 min, with the first fusion read being generated within five seconds even with a low tumor burden [15]. In the field of ALL research, the first application of third-generation sequencing technology led to the detection of BCR-ABL1 KD mutations in Ph-positive leukemia patients, revealing higher sensitivity and specificity than Sanger sequencing [16].

It was proposed a model for childhood B-ALL development that highlights early-life aneuploidy or oncogenic translocations as initiating events, followed by focal deletions and mutations promoting (pre-)leukemic fitness [17]. Most pediatric ALL subtypes exhibit a comparable number of putative driver gene alterations to adult cancers (4–5 per sample) [18]. NGS implementation in clinical practice will help understanding of the complex genomic relationship between molecular subtypes and their secondary alterations that drive leukemogenesis in B-ALL patients.

### 2.2. Whole Genome Sequencing

The current diagnosis of ALL does not incorporate WGS in most of the centers worldwide. However, the feasibility of incorporating WGS into genetic tests is growing, as many efforts have been made to overcome analytical challenges [19]. Indeed, some healthcare entities, such as the NHS Genomic Medicine Service and Genomic Medicine in Sweden, provide WGS for hematological and pediatric malignancies [20].

WGS is highly effective in identifying the rapidly increasing list of newly reported and cytogenetically cryptic abnormalities [21]. Some new molecular variants have emerged with WGS; the profile of *PAX5* rearrangements and the *ETV6::RUNX1*-like subtype have been characterized in more detail, and the detection of *DUX4* rearrangements has been markedly improved by a novel bioinformatics pipeline [22].

WGS significantly contributes to the definition of the complex genomic landscape underlying of these subtypes, highlighting some advantages over WTS, including the detection of focal copy number alterations (CNAs) and non-fusion gene rearrangements. A recent study investigated the suitability of using whole genome sequencing (WGS) as the sole diagnostic method to detect clinically relevant genomic aberrations in pediatric B-cell acute lymphoblastic leukemia, demonstrating a very high concordance between WGS findings and SoC results and allocation to the correct genetic subgroups in all cases [22].

In addition, WGS is able to detect lesions not routinely investigated in SoC (primary class-defining aberrations in the majority of B-other ALL samples including *DUX4-r*) and to allocate B-other ALL patients to one of the emerging genetic subgroups. WGS on B-other ALL patients from the UKALL14 trial assigned 88% of the cases called B-other to an established genetic subtype of ALL, with ~20% of the subtypes being assigned solely via the novel WGS workflows developed, revealing additional complex structural variants (IGH::CEBPA, IGH::ID4, and IGH::MIR125B1) missed by SoC methods [23].

Despite WGS detection of SVs, challenges persist due to short DNA and repetitive sequences [24,25]. Optical genome mapping (OGM) has emerged as a high-resolution, whole genome approach utilizing long, high-molecular-weight DNA, achieving read lengths over 200 kbp [26]. OGM simplifies the mapping of repetitive regions, enables chromosome arm coverage, and accurately detects small (500 bp) and larger (>30 kbp) SVs [27], demonstrating high concordance with molecular cytogenetics and SNP array analysis [28,29]. OGM identifies recurrently altered regions in B-ALL and novel regions missed by SNP array analysis, validated by long-read sequencing and/or RNA-seq [30]. However, standardized testing is essential before its integration into diagnostics, and parallel RNA and long-read sequencing are necessary to cover the full spectrum of genomic alterations.

### 2.3. RNA Sequencing

Transcriptome sequencing (RNA-seq) enhances B-ALL classification and risk stratification by identifying fusion genes, quantifying gene expression, and revealing rare transcripts often missed by WES or WGS, such as those caused by intronic mutations, splice-site mutations, or non-coding variants.

RNA-seq efficiently detects genetic rearrangements identified by traditional tests, covering both common fusions (*ETV6-RUNX1*, *TCF3-PBX1*, *BCR-ABL1*) and novel genetic rearrangements potentially influencing B-ALL pathogenesis, while sensitivity to a low tumor burden or lowly expressed fusions (like *KMT2A* rearrangements) and rearrangements in promoter/enhancer regions (e.g., IGH rearrangements) is limited [31].

RNA-seq improves the diagnosis of high-risk subtypes with a poor prognosis and resistance to standard chemotherapy. Among these, the detection of *IKZF1* deletion, associated with the worse B-ALL prognosis, especially if combined with the presence of the *BCR-ABL1* fusion gene [32], has been a challenge due to *IKZF1* deletion heterogeneity. Recently, researchers have developed a method to detect it using RNA-seq data [31]. In addition, a recent work has detected *DUX4* rearrangements through atypical *DUX4* gene expression and an alternative *ERG* exon, or gene expression clustering analysis [33].

Machine learning enhances acute leukemia diagnosis using RNA-seq gene expression data [34]. Currently, three alternative tools [ALLSpice [35], ALLSort [36], and ALLCatchR [37]] classify B-ALL subtype with RNA-seq based on GEP. Recently, MD-ALL (the molecular diagnosis of acute lymphoblastic leukemia), a user-friendly B-ALL classification platform, integrates gene expression profiling with mutation information for accurate classification, especially when GEP results are ambiguous or in conflict, making it valuable for clinical and research use [38].

It has been suggested that targeted RNA sequencing might substitute FISH and RT-PCR methods, simplifying the current diagnostic strategy for ALL, while providing the identification of both classical and modern subgroups of ALL, except for ploidy and copy number alterations [33].

RNA-seq integration in a clinical needs rapid and accurate detection to report clinically relevant alterations. RaScALL has emerged as a tool for rapidly screening RNA-seq data, providing comparable detection of clinically relevant ALL gene fusions, single-nucleotide variants, and small deletions and superior *DUX4r* detection at lower levels than alignment-based de novo variant calling tools, with shorter runtimes and lower memory requirements [39].

The German study group of the international AIEOP-BFM ALL 2017 trial performed a study on a consecutive cohort of 117 children with B-ALL in order to optimize the diagnostic standard workflow. In this study, results obtained with RNA sequencing analysis were compared to those obtained with conventional techniques such as RT-PCR, karyotyping, and FISH. This comparative analysis revealed overall coherence in 115/117 cases, except for one *AFF1-KMT2A* fusion undetected in the RNA sequencing and one *ETV6-RUNX1* fusion undetected in the conventional analyses. In conclusion, this study demonstrated that the combined application of RNA sequencing, FISH, and CGH+SNP array represent a reliable tool to detect all genetic markers necessary for risk stratification and will become the diagnostic standard workflow for B-ALL patients enrolled in the AIEOP-BFM ALL 2017 study. In addition, prospectively, data obtained with this diagnostic panel will support the elucidation and the identification of the genetic markers of pediatric ALL [40].

### 2.4. Proteomics

Proteomics plays a crucial role in analyzing the protein composition of leukemic cells, identifying differentially expressed proteins and providing insights into the disease mechanisms and biomarkers for diagnosis and therapeutic targets [41]. Identifying new biomarkers, such as cell surface proteins, can improve diagnosis, management, and targeted treatments. By examining the proteome, proteomics reveals dynamic protein states and their roles in cellular activities [42], offering insights that genomics alone cannot provide [43]. In fact, proteomics allows for comparison among disease stage samples, mainly using body fluids like CSF, PB, and BM [44,45]. Moreover, the proteomics studies are essential for understanding RNA transcription, alternative splicing, post-translational modifications, and their regulatory effects [46]. The mapping of the protein profile of leukemic cells has identified markers of leukemic aggressiveness, in accordance with the presence of t(12;21), typically indicating a good prognosis [47]. Despite significant advances in pediatric ALL, adult ALL remains challenging. A recent membrane proteome study has identified 67 differentially expressed protein spots in adult B-ALL patients, with 52 upregulated and 15 downregulated, including 5 proteins involved in energy metabolism [48]. A proteomic analysis of B-ALL samples from both pediatric and adult patients found elevated protein levels in specific pathways at relapse, like glycoloysis, phosphate pentose and metabolic pathways that might lead to chemo-resistance [49]. The analysis of plasma and urinary metabolites from 34 children revealed significant metabolic changes between ALL patients and controls, and between common B-ALL and pre-B ALL subtypes, reflecting treatment effects [50]. In addition, childhood ALL genomes show that relapses often emerge from subclonal outgrowths [51]. Retrospective analysis has found that targetable genomic variants and proteomic profiles persist throughout disease progression in pediatric ALL, showing a high correlation between drug response and variant-targeted therapies [52]. Together, these findings support the potential of proteomic approaches for diagnosis [53].

### 2.5. Farmacogenomics

Recent advances in genomic and transcriptomic profiling across the age spectrum enhance our knowledge of the differences in disease biology between children and adults and provide important insights into novel therapeutic targets. Subtype assignment can extend and refine the current standards of risk stratification, and current standards of care incorporate some molecular classification to identify patients at higher risk [31]. Drug sensitivity varies widely across different genetic subtypes, and this pharmacological heterogeneity is associated with treatment response and survival outcomes [54].

Favorable ALL subtypes, such as *ETV6-RUNX1* and hyperdiploidy, are more sensitive to L-asparaginase and glucocorticoids, whereas high-risk subtypes, such as *KMT2A*, *BCR-ABL1*, and *BCR-ABL1*-like, are more resistant [54]. Even *BCR-ABL1*-like ALL shows 73 times more resistance to asparaginase, 1.6 times more resistance to daunorubicin, and demonstrates poor sensitivity to glucocorticoids compared to standard-risk ALL [55,56]; de novo and acquired resistance to chemotherapy is known to be a major cause of treatment failure. In this context, TKIs like imatinib and dasatinib may target *ABL*-class fusion gene mutations. *EPOR* and *JAK2* rearrangements, along with mutations activating the JAK-STAT pathway like *CRLF2-R* might respond to JAK inhibitors such as ruxolitinib [57]. Crizotinib can target other rare kinase alterations in Ph-like ALL. FAK inhibitors could be effective against *NTRK3* and *PTK2B* fusion genes, and *TYK2* inhibitors may work for *TYK2* fusion genes. The effective role of target therapy in Ph-like ALL is still under evaluation in numerous phase II and III studies incorporating inhibitors into standard chemotherapy regimens [58,59,60]. Currently, other targets are under investigation for poor-prognosis ALL subtypes such as *KMT2A*-rearranged. This *KMT2A*-r-ALL displays relative resistance to corticosteroids and L-asparaginase chemotherapy. Nevertheless, it demonstrates sensitivity to cytosine arabinoside (Ara-C) and other nucleoside analogs. The sensitivity to Ara-C could be attributed to increased levels of human equilibrative nucleoside transporter 1 (hENT1) expression in *KMT2A*-rearranged ALL, while glucocorticoid resistance is thought to be mediated by the Src kinase-induced phosphorylation of annexin A2 [61,62,63]. However, despite the extended cytarabine inclusion in ALL chemotherapy regimens, the outcomes for *KMT2A*-r-ALL remain poor; this has prompted the development of new molecules targeting the *KMT2A* pathway such as menin inhibitors. Menin inhibitors disrupt the binding of *KMT2A* to menin, a crucial co-factor essential for the binding of the *KMT2A* complex to promoters of target genes, and maintain leukemogenesis specifically in leukemic cells [64]. Multiple clinical trials with these agents have been started with early results demonstrating clinical activity and promising benefits.

Given the genomic heterogeneity of childhood ALLs and the incomplete knowledge of their relationships with leukemia drug sensitivity, integrating pharmacotyping into sequencing approaches may enhance precision medicine.

## 3. Genetic Alterations in Pediatric B-ALL

Pediatric ALL includes different subgroups defined by chromosomal aberrations like abnormal chromosomal numbers, translocations, or other structural rearrangements. Before the introduction of next-generation sequencing (NGS) into clinical practice, 30% of B-ALL cases had inconclusive or absent subtype-defining abnormalities (denoted B-other), complicating treatment planning and disease monitoring for these patients. B-cell ALL is characterized by three main genetic alterations: point mutations, chromosomal aneuploidy, and rearrangements that encode chimeric transcription factors or misregulate oncogenes [65] (Table 2).

**Aneuploidy and copy number gain**. Aneuploidy is a hallmark of ALL. High hyperdiploidy (>50 chromosomes), found in ~30% of pediatric B-ALL cases, correlates with a favorable outcome and involves Ras pathway mutations and chromatin modifiers such as *CREBBP*, and has favorable outcomes [106,107]. Conversely, low hypodiploidy (31–39 chromosomes), rare in children (1%) but increasing with age (>10% of adults), involves loss-of-function mutations in *TP53* (91% of both pediatric and adult cases), *IKZF2* (53% of pediatric; 36% of adult cases), *RB1* (41% of pediatric; 19% of adult cases), histone modifiers (60%), and *CDKN2A/B* (20%) [108,109]. Near haploidy (24–30 chromosomes), observed in ~2% of childhood B-ALL cases, associates with Ras mutations (71% of cases), *NF1* (44%), histone modifiers (64%), mainly *CREBBP* (32%), *CDKN2A/B* (20%), the 6p22 histone gene cluster (19%), *IKZF3* (13%), and *PAG1* (10%). Unfortunately, both low-hypodiploid and near-haploid B-ALL are linked to unfavorable outcomes [68,69]. The intrachromosomal amplification of chromosome 21 (*iAMP21*), an abnormal version of chromosome 21 containing multiple regions of gain, amplification, inversion and deletion, is most common in older children and is associated with a poor prognosis [72].

Several molecular pathways and gene expressions have been identified in association with B-ALL, and these will be detailed below.

***ETV6-RUNX1/ETV6-RUNX1*-like ALL**. The *ETV6-RUNX1* fusion is the most common translocation in childhood B-ALL resulting from t(12;21)(p13;q22), is often cryptic on cytogenetic analysis, and is associated with a good prognosis. *ETV6-RUNX1* patients exhibit different aberrations (up to 60) highlighting B-ALL heterogeneity, impacting treatment response, and complicating management [74,75]. *ETV6-RUNX1*-like ALL (found in almost 3% of pediatric ALL cases) shares a similar gene expression profile and immunophenotype (CD27+, CD44 low/negative) to *ETV6-RUNX1* ALL, with a relatively favorable prognosis, but it is characterized by *ETV6* and *IKZF1* aberrations [76].

***BCR-ABL1/BCR-ABL1*-like ALL** (Philadelphia-like ALL, Ph-like ALL). The Philadelphia chromosome (Ph), resulting from t(9;22)(q34;q11.2), encodes the *BCR-ABL1* fusion protein and occurs with a higher incidence in adults (25–30%) than in children (3–5%) affected by B-ALL. The prognosis of *BCR-ABL1*-positive adult and pediatric B-ALL patients is extremely poor. However, the addition of tyrosine kinase inhibitors (TKIs) (e.g., imatinib, dasatinib, etc.) to intensive chemotherapy has significantly improved outcomes for BCR-ABL1-positive ALL patients. There are three variants of the *BCR-ABL1* oncogenic fusion protein with different sizes (p190, p210, and, rarely, p230), depending on the breakpoint heterogeneity on the *BCR* gene. The variant involving breakpoints between exons 13 and 14 or exons 14 and 15 of *BCR* (major BCR) is frequently observed in chronic myelogenous leukemia (CML) and encodes a 210 kDa fusion protein [110], while the variant involving a breakpoint between exons 1 and 2 of *BCR* (minor BCR) encodes a 190 kDa fusion protein and is frequently observed (90%) in BCR-ABL1-positive pediatric B-ALL. These fusions lead to the constitutive activation of the tyrosine kinase function with the activation of multiple signaling pathways, increased cell proliferation, and impaired differentiation and adhesion [77]. Additional secondary abnormality, such as *IKZF1* deletions, is found in about 84% of *BCR-ABL1*-positive B-ALL cases and is linked to chemotherapy resistance and a higher risk of relapse [111].

*BCR-ABL1*-like ALL shares a similar gene expression profile with Ph-ALL but lacks the BCR-ABL1 rearrangement [55,79]. It is associated with an unfavorable prognosis and a high risk of relapse [80], affecting 15% of children, 20–25% of adolescents/young adults and adults. *BCR-ABL1*-like ALL involves different genetic alterations: ABL-class gene fusions (*ABL1*, *ABL2*, *CSF1R*, *PDGFRB*, and *PDGFRA*), found in about 10% of patients, and *CRLF2* rearrangements present in more than 50% of Ph-like cases. Other alterations include mutations in the *JAK2* (9.0%), *EPOR* (5.7%) [81], and RAS pathway genes, such as *KRAS*, *NRAS*, *NF1*, *PTPN11*, *CBL1*, and *BRAF* (4%) [82]. However, about 5% to 10% of patients with *CRLF2*-rearranged (*CRLF2-R*) ALL, particularly children, have distinctly different gene expression profiles that lack genetic alteration activating tyrosine kinase signaling [81]. Patients with translocations involving tyrosine kinases have improved clinical outcomes with remarkable responses to TKI therapy.

***TCF3* ALL**. The *TCF3*-HLF ALL is a rare (<1%) B-ALL subtype, often associated with older age and a poor outcome. It results from the t(17;19)(q22;p13) translocation, creating a fusion protein that combines the transactivation domain of *TCF3* (*E2A*) with the DNA-binding and dimerization domains of *HLF* (hepatic leukemia factor), a basic leucine zipper transcription factor belonging to the PAR family. This fusion protein regulates apoptosis-related genes in lymphoid progenitors [112,113]. Two major fusion breakpoints between *TCF3* and *HLF* (exon 13 of *TCF3* and exon 4 of *HLF*, and exon 12 of *TCF3* and exon 4 of HLF) lead to distinct clinical presentations [114]. Chemoresistance in *TCF3-HLF* ALL is linked to upregulated RAS and BCL-2 pathways, increased P-glycoprotein expression, and ABC multi-drug resistance transport proteins [84]. Additionally, the *TCF3-PBX1* (*E2A-PBX1*) fusion protein, resulting from the t(1;19)(q23;p13) translocation and its unbalanced variant der(19)t(1;19)(q23;p13), downregulates *TCF3* encoded transcription factors E12 and E47, which regulate the early lymphoid development. This subtype occurs in 5% of pediatric B-ALL cases, with an intermediate prognosis due to advanced treatments. *TCF3-PBX1*-positive patients exhibit a characteristic B-cell immunophenotype and gene expression profile, constituting a separate entity among ALL patients. The WHO classifies *TCF3-PBX1*-positive leukemia as a distinct entity among B-lymphoblastic leukemias [115]. The t(1;19)(q23;p13) translocation encoding *TCF3-PBX1* is more frequent in African-Americans and is associated with an increased risk of central nervous system (CNS) involvement [85].

***KMT2A* ALL**. MLL (mixed-lineage leukemia) (actually renamed Lysine K-specific Methyltransferase 2A or *KMT2A*) gene rearrangements at 11q23 are found in 80% of infant B-cell ALL (0–2 years old) and in 10% of childhood B-cell ALL, often leading to poor outcomes due to drug resistance, even with modern chemotherapy and hematopoietic stem cell transplantations [116]. *KMT2A*-ALL patients commonly present with central nervous system involvement at diagnosis, a rapid onset, and hyperleukocytosis [86]. The *KMT2A* gene can rearrange with more than 80 partner genes (i.e., *AF4*, *AF9*, *ENL*, *ELL*, and *AF10*), resulting in common translocations like t(4;11)(q21;q23) (about 50% of MLL rearrangements), t(9;11)(p22;q23), t(11;19)(q23;p13.3), and t(10;11)(p13-14;q14-21) encoding *MLL-AFF1*(*AF4*), *MLL-MLLT3*(*AF9*), *MLL-ENL*, and *MLL-MLLT10*(*AF10*), respectively [86,87]. These rearrangements produce fusion proteins that disrupt the normal histone methylation involved in the regulation of *HOXA* and *MEIS1* expression, leading to leukemic transformation by altering chromatin structure and epigenetic regulation [88].

***IKZF1* ALL**. Mutations in the *IKZF1* gene, mainly deletions and rarely point mutations, are observed in high-risk B-ALL, occurring in 80% of *BCR-ABL1*-positive and 70% of *BCR-ABL1*-like cases. *IKZF1* gene encodes for Ikaros, a transcription factor essential for lymphoid development and differentiation [3,117]. These deletions act as dominant negative mutations, inhibiting the function of the wild-type Ikaros and blocking cell differentiation. This suggests that the impairing of Ikaros activity contributes to B-ALL leukemogenesis [89]. *IKZF1* mutation/deletion predicts chemotherapy resistance, high relapse risk, and poor clinical outcomes [90]. A study of 991 B-ALL patients in the AIEOP-BFM ALL 2000 trial identified a high-risk group, *IKZF1plus*, characterized by the co-occurring of *IKZF1* deletions with *PAX5*, *PAR1*, *CDKN2A*, or *CDKN2B* deletions (excluding *ERG* deletion). This MRD-dependent B-ALL profile does not respond to the current AIEOP-BFM treatment, indicating a very poor prognosis [91].

***CRLF2* ALL**. Rearrangements or mutations in cytokine receptor-like factor 2 (*CRLF2*), located at the pseudo-autosomal region (*PAR1*) at Xp22.3/Yp11.3, occur in up to 7% of B-ALL cases and in almost 50% of Down syndrome-associated (DS-associated) B-ALL cases. These alterations are also found in up to 50% of high-risk B-ALL cases such as *BCR-ABL1*-like ALL [92,93]. The rearrangements are either rearrangement into IGH@-CRLF2 or deletion immediately upstream of *CRLF2*, both resulting in the overexpression of *CRLF2* on the cell surface. The p.Phe232Cys mutation occurs in the transmembrane domain of *CRLF2* resulting in overexpression and dimerization of the receptor [94]. *CRLF2* alterations are associated with activating mutations in the Janus kinase genes *JAK1* and *JAK2* which most commonly disrupt p.Arg683 in the pseudokinase domain of *JAK2*, resulting in cytokine-independent proliferation in cultured cells. *CRLF2* rearrangements are associated with *IKZF1* alteration, *JAK* mutation, and poor outcomes in non-DS associated ALL [92,95].

***DUX4* ALL**. *DUX4* encodes for a double-homeobox transcription factor and its translocation to the immunoglobulin heavy-chain locus (IGH) occurs in 5–10% of B-ALL cases resulting in a distinctive gene expression profile and immunophenotype (CD2±, CD371+). *DUX* alterations involve not only *DUX4* rearrangement and its overexpression but also *ERG* gene deregulation or deletion, marking a B-progenitor ALL subtype (up to 7%). *DUX4* alterations often coincide with deletions in *IKZF1*, *PAX5*, and *CDKN2A*/*CDKN2B* and the activation of mutations in *NRAS*, *KRAS*, *MYC*, *MYCBP2*, *MGA*, and *ZEB2*. Despite *IKZF1* deletion, typically an adverse prognostic factor in 40% of cases, *DUX4*-rearranged B-ALL exhibits a favorable outcome [96,97].

***ZNF384/ZNF384*-like ALL**. *ZNF384*, rearranged in 6% of childhood B-ALL cases, and *ZNF362*, often rearranged in ZNF384-like cases, encodes C2H2-type zinc-finger transcription factors and rearranges with genes encoding N-terminal transcription factors (e.g., *TAF15* and *TCF3*) or chromatin modifiers (most commonly EP300, but also *CREBBP*, *SMARCA2*, and *ARID1B*) [98,99].

***MEF2D* ALL**. *MEF2D* (myocyte enhancer factor 2D) rearrangements, occurring in 4% of children (median: 14–15 years) and up to 10% of adults with B-ALL, associate with a peculiar immunophenotype (CD10−, CD38+) and a poor prognosis, but increased HDAC9 expression suggests potential histone deacetylase inhibitor therapy [100,101].

***NUTM1* ALL**. NUTM1 (nuclear protein in testis midline carcinoma family 1) rearrangement with transcription factors and epigenetic regulators (*ACIN1*, *BRD9*, *CUX1*, *IKZF1*, *SLC12A6*, and *ZNF618*) occurs in 1–2% of childhood B-ALL cases and has a good prognosis [102].

***PAX5* ALL**. Pax-5 (paired box protein), a key transcription factor, modulates B-cell dynamics (development, differentiation, migration, and proliferation). Aberrant Pax-5 expressions, prevalent in over 90% of pediatric B-ALL cases, can trigger leukemic transformation in early B-ALL [118] (Tiacci et al., 2004). Pax-5 fusion with other proteins, such as Janus kinase (Jak) 2 promotes B-cell proliferation through the Jak-STAT signaling pathways [119].

Two B-ALL subtypes are associated with *PAX5* alterations with an intermediate to favorable prognosis, *PAX5* p.Pro80Arg mutation (PAX5 P80R) [105] and PAX5-altered (PAX5alt), that includes rearrangements (commonly with *ETV6* or *NOL4L*), mutations, or intragenic amplifications [13,103,104].

## 4. Conclusions and Future Perspectives

Over the past few years, significant progress has been made in the understanding of B-ALL biology and genetics thanks to gene expression profiling and genome-wide sequencing analyses. In particular, this progress has proven especially useful for classifying B-ALL patients with different risk profiles, identifying new therapeutic targets, and ultimately improving overall clinical outcomes. Advancements in next-generation sequencing (NGS) have made it possible to discover novel genetic groups and pathways in ALL and have revolutionized the understanding of tumor genomic heterogeneity, influencing the selection of molecular biomarkers and clinical decisions in precision therapies. Genome-based analysis, such as exome sequencing and whole-genome sequencing, and transcriptomic data on extensive patient cohorts have enriched insights into the pathogenesis, progression, prognosis, risk stratification, and therapies for ALL. Standardizing clinical NGS workflows is essential for global guidelines on new diagnoses and minimal residual disease (MRD) monitoring. The implementation of NGS in clinical practice is one of the most important next goals since it may replace traditional methods. This highly sensitive technique, that can reach up to 10^−6^, allows for the precise quantification of residual leukemic cells beyond the limits of traditional flow cytometry or PCR-based methods [120]. Moreover, it facilitates tailored therapeutic decision-making, including the de-escalation of treatment intensity in low-risk patients, reducing the long-term toxicities associated with conventional therapy. For patients undergoing hematopoietic stem cell transplantation, NGS-MRD provides a robust predictive marker for post-transplant relapse, guiding the timing and modality of transplantation and post-transplant surveillance strategies [121].

Although genomic analyses have shed light on the genetic landscape of pediatric ALL, the determination of the underlying changes at a protein level is still a challenge. In fact, changes in protein expression cannot be deduced completely by the analysis of the genome. Proteomics analyzes protein structure, expression, and modifications offering the opportunity to discover novel biomarkers and druggable targets. In recent years, omics approaches have revealed a high number of biomarkers, although still very few of them have been validated to support their use in the clinic. This will require validation initiatives and collaborations at national and international levels to offer new perspectives for innovation in ALL diagnostics and therapeutics. Omics studies require researchers with different expertise, including data managers and bio-informaticians, and the incorporation of more clinicians into research teams should be crucial for the transfer of clinical innovation into practice.

Finally, we can conclude that, in the era of personalized medicine, the search for and implementation of novel biomarkers with prognostic and predictive value, as well as more efficacious targeted therapeutic agents, represent the current challenge in the implementation of molecular diagnosis and subclassification in the clinical practice and care for B-ALL.

## Figures and Tables

**Table 1:** Factors that influence the prognosis of B-ALL.

	Good Prognosis	Bad Prognosis
**Gender**	Females	Males
**Age**	<30 years	<1 year >65 years
**Ethnicity**	Caucasians	African Americans, Asians, and Hispanics
**Cytogenetic features**	Hyperdiploidy > 50 (51–65 chromosomes) t(12;21) t(1;19)	t(9;22) (more common among adults) t(4;11) (4% of cases and is most common in infants under 12 months) t(8;14)(q24.1;q32) t(8;14) t(2;8) t(8;22) t(5;14) hypodiploidy (<46 chromosomes) haploidy triploidy (66–68 chromosomes) del (17p) t(11q23) t(1;19) in relapse
**Other factors**	White blood cell count <25,000/mcL (<25 × 109/L) or <50,000/mcL (<50 × 109/L) No central nervous system involvement at the time of diagnosis	high initial white blood cell count over 100 × 109/L; involvement of the nervous system and other organs; slow response to initial treatment; presence of minimal residual disease after treatment

**Table 2:** Main genetic alterations in B-ALL.

Genetic Alterations	Frequency in Childhood B-ALL	Prognosis	Targeted Therapy	References
High hyperdiploidy (51–65 chromosomes)	30%	Good	None	[66,67]
Near Haploidy (24–31 chromosomes)	1–2%	Poor	Potential use of PI3K inhibitors	[68,69]
Low Hypodiploidy (32–39 chromosomes)	1–2%	Poor	Potential use of PI3K inhibitors	[69,70,71]
*iAMP21* (intrachromosal amplification of chromosome 21)	1.5–2%	Intermediate	None	[72,73]
*ETV6::RUNX1* (gene traslocation) t(12;21)(p13;q22)	25%	Good	None	[74,75]
*ETV6::RUNX- like* (gene translocation) absence of *ETV6-RUNX1* fusion; mutations in both *ETV6* and *IKZF1*	2–3%	Poor	None	[76]
*BCR::ABL1* (Philadelphia chromosome) t(9;22)(q34;q11)	3–5%	Poor	Tyrosine kinase inhibitors	[77,78]
Ph-like ALL (Gene fusions)	15%	Poor	TKI, JAK2 inhibitors, JAK1/JAK3 inihibitors, TYK2 inhibitors, Crizotinib, MEK inhibitors, FAK inhibitors, FLT3 inhibitors	[55,79,80,81,82]
*TCF3::HLF* (gene traslocation) t(17;19)(q22;p13)	<1%	Poor		[83,84]
*TCF3::PBX1* (gene traslocation) t(1;19)(q23;p13)	6%	Intermediate	Dasatinib, Ruxolinitib	[85]
*KMT2A* (gene rearrangements 11q23) (t(11q23))	80% (infant), 10% (childhood)	Poor		[86,87,88]
*IKZF1* (deletion/point mutation/gene fusions) t(3;7)(q27;p12)	16–27%	Poor	None	[89,90,91]
*CRLF2* (gene fusions/point mutation) t(X;14)(p22;q32) or t(Y;14)(p11;q32)	5%	Poor	Potential use of JAK inhibitors	[92,93,94,95]
*DUX4* (gene fusions) t(4;19)(q35;q13)	4–7%	Good	Potential	[96,97]
*ZNF384* (gene fusions) t(12;17)(p13;q21)	3–5%	Intermediate	FLT3	[98,99]
*MEF2D* (gene fusions)	4%	Poor	HDAC inhibitors staurosporina, Venetoclax	[100,101]
*NUTM1* (gene fusions)	1%	Good	Bromodomain inhibitors	[102]
*PAX5* (gene fusion/deletion/amplification) t(9;22)(p13;q13)	7–10%	Intermediate	Tyrosine kinase inhibitors (NRAS, KRAS, and FLT3)	[13,103,104]
*PAX5 * Hot-spot mutation (Pax5: p.Pro80Arg)	3–4%	Intermediate	Potential use of Ros, JAK/STAT, FLT3, BRAF, and PIK3CA inhibitors	[105]
